# Evaluation of Immunogenicity to Three Doses of the SARS-CoV-2 BNT162b2 mRNA Vaccine in Lung Transplant Patients

**DOI:** 10.3390/vaccines10101642

**Published:** 2022-09-30

**Authors:** Mariasilvia Guardiani, Maria Antonella Zingaropoli, Francesco Cogliati Dezza, Anastasia Centofanti, Carolina Carillo, Eeva Tortellini, Federica Dominelli, Anna Napoli, Cosmo Del Borgo, Aurelia Gaeta, Federico Venuta, Vincenzo Vullo, Miriam Lichtner, Maria Rosa Ciardi, Claudio Maria Mastroianni, Gianluca Russo

**Affiliations:** 1 Department of Public Health and Infectious Diseases, Sapienza University of Rome, 00185 Rome, Italy; 2 Department of General and Specialistic Surgery “Paride Stefanini”, Sapienza University of Rome, 00161 Rome, Italy; 3 Department of Molecular Medicine, Sapienza University of Rome, 00161 Rome, Italy; 4 Infectious Diseases Unit, Santa Maria (SM) Goretti Hospital, Sapienza University of Rome, 04100 Latina, Italy; 5 Department of Neurosciences, Mental Health, and Sense Organs, NESMOS, Sapienza University of Rome, 00189 Rome, Italy

**Keywords:** lung transplanted patients, humoral response, T-cell response, BNT162b2 mRNA vaccine, anti-spike antibody, flow-cytometry

## Abstract

The aim of the study was to explore the humoral and T-cell response in lung transplant (LuT) patients. Two-time points were considered, before (T0) and after (Tpost) the third dose of the BNT162b2 mRNA vaccine, comparing LuT with healthy donors (HD). LuT patients showed a lower serologic response against SARS-CoV-2 compared with HD at both time-points (*p* = 0.0001 and *p* = 0.0011, respectively). A lower percentage of IFNγ+orIL2+orTNFα+CD4+ and CD8+ T-cells LuT patients was observed in LuT patients compared with HD at T0 (CD4+: *p* = 0.0001; CD8+: *p* = 0.0005) and Tpost (CD4+: *p* = 0.0028; CD8+: *p* = 0.0114), as well as in the percentage of IFNγ+IL2+TNFα+CD4+ T-cells (T0: *p* = 0.0247; Tpost: *p* = 0.0367). Finally, at Tpost, a lower percentage of IFNγ+IL2+TNFα+ CD8+ T-cells in LuT patients compared with HD was found (*p* = 0.0147). LuT patients were stratified according to the lowest cut-off value for the detection of a humoral response (4.81 BAU/mL) at T0, into responder (R) and non-responder (NR) groups. In the R group, no differences in the percentage of IFNγ+or IL2+orTNFα+ and IFNγ+IL2+TNFα+CD4+ and CD8+ T-cells compared with HD at both time-points were observed. Otherwise, in the NR group, lower percentages of IFNγ+IL2+TNFα+CD4+ T-cells compared with the R group (T0: *p* = 0.0159; Tpost: *p* = 0.0159), as well as compared with the HD, at both time-points, were observed (T0: *p* = 0.0064; Tpost: *p* = 0.0064). These data seem to confirm that some LuT patients can mount cellular responses even in the absence of a positive humoral response (>33.8 BAU/mL), although this cellular response is dysfunctional and partially detrimental.

## 1. Introduction

Every year, 4500 lung transplants (LuT) are carried out over the world, with about 2000 in Europe and just over 100 in Italy [1].

Several studies have shown a higher risk of severe COVID-19 (Coronavirus Disease) among immunocompromised patients, but the impact on solid organ transplant (SOT) patients remains debated [2,3,4]. It is well known that LuT patients are at high risk of severe COVID-19 because the respiratory system is the main target of severe acute respiratory syndrome coronavirus-2 (SARS-CoV-2) infection [5,6]. Furthermore, LuT patients may be more vulnerable than other SOT patients because of the higher burden of chronic immunosuppression therapy [1,5]. Vaccination represents a valuable tool for the prevention of severe disease in fragile patients, including SOT patients [5,6].

Among the vaccines based on different technologies, the BNT162b2 mRNA vaccine (Comirnaty^®^) has been widely used in Italy, as well as for the immunization of fragile and immunosuppressed individuals [7,8,9,10,11]. Several studies have reported that mRNA-based vaccines induce a robust and protective humoral [9,12] and cellular response against the Spike SARS-CoV-2 protein in immunocompetent subjects, leading to a lower morbidity and mortality for COVID-19 [7,11]. However, as reported in the longitudinal evaluation between the first and the second dose of mRNA SARS-CoV-2 vaccine in SOT patients performed by Boyarsky et al. [13], a high rate of SOT patients included in the study showed a detectable antibody response after the second dose, although low antibody levels were observed in those patients without a detectable response after the first dose.

Following antigenic stimulation by vaccination, the development of long-term protective immunity depends on a range of immunological events, including the activation, proliferation, differentiation, and coordination of the humoral and T-cell response [12]. Specifically, for COVID-19, analysis of the humoral and T-cell response underlines the importance of cellular immunity in immunocompromised patients, including SOT patients [4]. Indeed, despite the absence of an antibody response, most of the immune responses were associated with Spike-specific T-cells [5]. Finally, for most vaccines, booster doses are recommended to induce long-lasting protection from infection as repeat immunogenic stimulations increase the intensity and durability of the cellular immunity [12].

To better clarify the immune response to the BNT162b2 mRNA vaccine in LuT patients, the aim of the study was to investigate the humoral and T-cell response at different time-points (before and after booster dose), with a focus on the phenotype of the T-cell response.

## 2. Materials and Methods 

### 2.1. Study Participants and Surveillance

An observational, monocentric, prospective study including adult LuT patients enrolled between October 2021 and March 2022 was conducted. As a control group, we enrolled healthy donors (HD), matched for age and gender. The inclusion criteria for both LuT patients and HD were: (I) being >18 years old, (II) having already received two mRNA BNT162b2 vaccine doses of anti-SARS-CoV-2, and (III) no evidence of previous SARS-CoV-2 infection. 

Both groups were tested for the humoral and T-cell response at two time-points, two months before (T0) and after (Tpost) the third vaccine dose administration. Specifically, at T0, blood samples were taken on the same day as the third dose of the mRNA BNT162b2 vaccine, before the administration of the latter.

All LuT patients were evaluated for demographics, comorbidities, basic laboratory findings, months elapsed from transplant, immunosuppression therapy, and SARS-CoV-2 vaccination timing.

### 2.2. Determination of SARS-CoV-2 Anti-S and Anti-N

For each group, evaluation of the humoral response was performed at both time-points. The serum samples collected were evaluated with The DiaSorin Liaison SARS-CoV-2 TrimericS IgG (DiaSorin S.p.A, Saluggia, VC, Italy) chemiluminescence immunoassay (CLIA), which detects SARS-CoV-2 spike S1/S2 protein specific IgG antibody levels. The performance of the sensitivity and specificity were reported according to the manufacturer’s instructions. The levels of anti-SARS-CoV-2 IgG antibodies were expressed using the World Health Organization International Standard (NIBSC code. 20/268) binding antibody unit (BAU/mL). A positive serologic response was defined as having detectable IgG antibodies against SARS-CoV-2 over 33.8 BAU/mL. The lower and upper detection limits of the assay were 4.81 BAU/mL and 2080 BAU/mL, respectively. 

To confirm no previous SARS-CoV-2 infection, the presence of the anti-N SARS-CoV-2 antibody was determined using the KT-1032 EDITM Novel Coronavirus COVID-19 IgG Enzyme Linked Immunosorbent Assay (ELISA) Kit (Epitope Diagnostics, Inc. 7110 Carroll Rd, San Diego, CA, USA) and it was performed according to the manufacturer’s instructions. The average value of the absorbance of the negative control was less than 0.25, and the absorbance of the positive control was not less than 0.30. 

### 2.3. Stimulation of T-Cell Using SARS-CoV-2 Peptides Libraries

To evaluate the T-cell specific response, peripheral blood mononuclear cells (PBMCs) were isolated by density gradient centrifugation using Ficoll-Paque PLUS (Sigma-Aldrich, Saint Louis, MO, USA) and then cryopreserved in cell recovery media containing 10% dimethyl sufoxide (DMSO) supplemented with heat inactivated Fetal Calf Serum (FCS). Finally, PBMCs were stored at −196 °C until they were used.

The T-cell specific response was assessed using multiparametric flow cytometry after overnight stimulation with SARS-CoV-2 peptide libraries, as previously shown [14]. Briefly, 1 × 10^6^ PBMCs resuspended in 200 μL of RPMI were incubated overnight at 37 °C and 5% CO_2_. For each patient, a negative (unstimulated condition) and positive control (phytohemagglutinin (PHA) condition, 5 μg/mL) was also included. Brefeldin A at a final concentration of 5 μg/mL was added in the culture after 1 h of incubation. 

After overnight stimulation, PBMCs were incubated for 30 min with Fixable Viability Dye and thy were washed in stain buffer (SB) containing 1% FCS. PBMCs were stained with the appropriate mix of monoclonal antibodies (Pacific Blue-conjugated anti-CD45, APC-conjugated anti-CD8 and APC-Cy7-conjugated anti-CD4) and incubated in darkness at 4 °C for 20 min. Then, the red blood cells were lysed using the lysing solution (BD Biosciences, Franklin Lakes, NJ, USA), in darkness at room temperature for 20 min (BD Biosciences, Franklin Lakes, NJ, USA). Fix/Perm solution (BioLegend, San Diego, CA, USA) was used prior to intracellular staining (FITC-conjugated anti-IFNγ, PerCp-Cy5.5-conjugated anti-TNFα, and PE-Cy7-conjugated anti-IL2), according to the manufacturer’s instructions. The cells were washed once in Perm wash solution (BioLegend, San Diego, CA, USA) according to the manufacturer’s instructions. All the antibodies were from BioLegend. Finally, the cells were fixed in Phosphate-Buffered Saline (PBS) containing 0.5% formaldehyde (Sigma-Aldrich, St. Louis, MO, USA).

**Figure 1:**
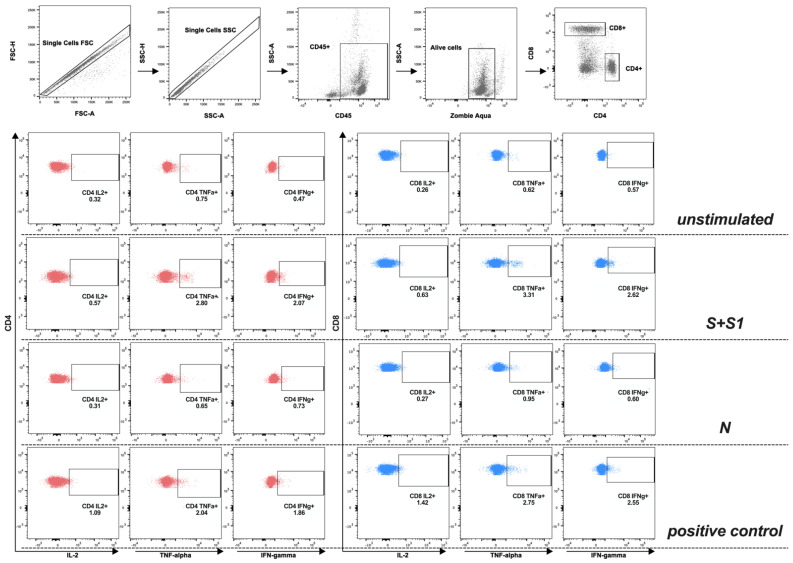
Representative flow cytometry gating strategy showing cytokine-producing cells among CD4+ or CD8+ T-cells. After single cells in forward scatter area (FSC-A) versus height (H) and side scatter area (SSC-A) versus H were plotted, CD45+ cells were identified. Then, dead cells were excluded. Finally, CD4+ and CD8+ T-cells were identified, as well as IL2, IFNγ, and TNFα. S+S1: spike glycoprotein; N: nucleocapsid phosphoprotein.

The stained samples were acquired using the MACSQuant Flow Cytometer (Miltenyi Biotec, Bergisch Gladbach, Germany) and were analyzed using FlowJo™ v10.8.1 software. The gating strategy for analysis of the antigen-specific T-cells is illustrated in Figure 1. The cytokine background obtained from the negative condition (unstimulated) was subtracted from the stimulated ones. As previously shown [15], the co-expression of cytokines was analyzed via Boolean gating using FlowJo™ v10.8.1. The display and analysis of the different cytokine combinations was performed with SPICE v6.1.

We labelled T-cells producing at least one of the three cytokines as IFNγ+orIL2+orTNFα+ and those that simultaneously producing all three cytokines as IFNγ+IL2+TNFα+.

### 2.4. Statistical Analysis

Statistical analyses were performed using GraphPad Prism v.9 for macOS. Two-tailed *p* ≤ 0.05 was considered statistically significant. Data are represented as median with interquartile range (IQR). The nonparametric comparative Mann–Whitney test and the nonparametric Kruskal–Wallis’s test with Dunn’s post-test were used for comparing the medians between groups. Longitudinal evaluation of anti-S levels and percentages of T cells producing IFNγ+orIL2+orTNFα+ and IFNγ+IL2+TNFα+ was performed using the nonparametric Wilcoxon test. Correlations between quantitative data were assessed using the non-parametric Spearman test. Linear correlation was evaluated using the regression test. Distributions of different cytokine combinations were performed by the nonparametric Wilcoxon rank test using SPICE, distributed by the National Institute of Allergy and Infectious Diseases, NIH.

## 3. Results 

### 3.1. Characteristics of Study Population, Clinical Presentation, and Laboratory Findings

The demographic and clinical features of the study population are reported in Table 1. Nine LuT adult patients (two female and seven male) with a median age (IQR) of 56 (46–62) years and nine HD (two female and seven male) with a median age (IQR) of 50 (47–58) years were enrolled. Among the LuT patients, the underlying disease that led to the lung transplant was emphysema (4/9, 44.4%), idiopathic pulmonary fibrosis (2/9, 22.2%), histiocytosis X (2/9, 22.2%), or bilateral congenital bronchiectasis (1/9, 11.1%). Overall, 45% (4/9) of LuT received a bilateral lung transplant, whereas the remaining 55% (5/9) received a monolateral lung transplant. As reported in Table 1, the median time elapsed from the transplant was 24 months. The antirejection chronic immunosuppressive therapy was a combination of a calcineurin inhibitor, mycophenolate mofetil (MMF), and prednisone. All the LuT patients were receiving low-dose steroid and calcineurin inhibitors treatments at both vaccination time-points, while 66% (6/9) were also treated with mycophenolate (Table 1). 

Overall, all LuT patients had at least one comorbidity and the most common were arterial hypertension (55%), diabetes mellitus (44%), cardiopathy (22%), and dyslipidemia (11%) (Table 1). No graft rejection in LuT patients was observed during the study period.

No adverse events among LuT patients and HD after receiving the third dose of the BNT162b2 mRNA vaccine were observed.

Finally, the median value (IQR) in days between the second dose of the vaccine and T0 for LuT patients and HD were 177 (175–182) and 179 (177–185), respectively.

### 3.2. Vaccination-Induced Humoral Response

Overall, all of the enrolled LuT patients and HD had a negative SARS-CoV-2 N-protein IgG serology test at both time-points. 

**Figure 2:**
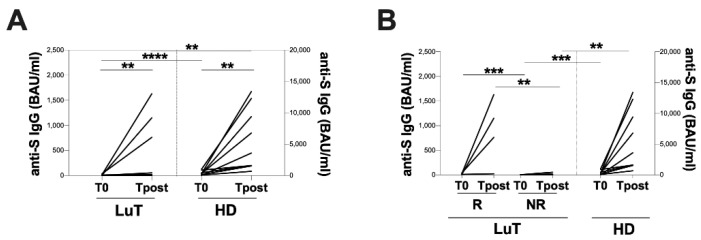
Evaluation of vaccination-induced humoral response. (**A**) Anti-S IgG titer was evaluated in the LuT and HD groups at T0 and at Tpost. Longitudinal evaluation was performed using Wilcoxon test. For each time-point, differences between LuT and HD were evaluated using the nonparametric Mann–Whitney test. (**B**) Stratification of LuT patients into R and NR, according to the anti-S IgG titer at T0. Longitudinal evaluation was performed using Wilcoxon test. Each group of LuT patients was compared to the respective HD time-points using the nonparametric Kruskal–Wallis’s test with Dunn’s post-test. T0: before the third dose of vaccine; Tpost: two months after the third dose; LuT: lung transplants; HD: healthy donors; R: responders; NR: non-responders; BAU: binding antibody unit. ****: *p* < 0.0001; ***: 0.0001 < *p* < 0.001; **: 0.001 < *p* < 0.01.

At both time-points, LuT patients showed significantly lower IgG antibodies against SARS-CoV-2 titer compared with HD (T0: 4.8 [4.8–16.2] and 320 [124.1–662.0] BAU/mL, respectively, *p* < 0.0001; Tpost: 23.0 [7.3–956.0] and 3590 [1575–10850] BAU/mL, respectively, *p* = 0.0011) (Figure 2A). 

At T0, 88.9% (8/9) of LuT patients had a serologic response against SARS-CoV-2 under the cut-off value of 33.8 BAU/mL, according to the manufacturer’s instructions. Conversely, at Tpost, 55.6% (5/9) of LuT patients showed a serologic response against SARS-CoV-2 under the cut-off value.

The longitudinal evaluation of IgG antibodies against the SARS-CoV-2 titer in LuT patients showed a significant increase at Tpost compared with T0 (23.0 [7.3–956.0] and 4.8 [4.8–16.2] BAU/mL, respectively; *p* = 0.0039) (Figure 2A). 

LuT patients were further stratified into two groups: responder (R, n = 4), including those patients who had a detectable serologic response against SARS-CoV-2 (cut-off > 4.81 BAU/mL), and non-responder (NR, n = 5), including those patients who had an undetectable serologic response against SARS-CoV-2 (cut-off < 4.81 BAU/mL). According to this stratification, at T0, 44.4% (4/9) of LuT patients showed detected IgG antibodies against SARS-CoV-2 titer (4.8 [4.8–16.2] BAU/mL), albeit at low levels. Conversely, at Tpost, all LuT patients showed detected IgG antibodies against SARS-CoV-2 titer (23.0 [7.3–956.0] BAU/mL, respectively, *p* = 0.0294). 

At both time-points, the R group showed a statistically significant higher IgG antibodies against the SARS-CoV-2 titer compared with the NR one (T0: 16.2 [8.5–31.7] and 4.8 [4.8–4.8] BAU/mL, respectively, *p* = 0.0079; Tpost: 956.0 [207.8–1510] and 9.4 [5.0–36.0] BAU/mL, respectively, *p* = 0.0317) (Figure 2B). No significant differences between the R group and HD at both time-points were observed (Figure 2B). Conversely, the NR group showed significant lower anti-S titer compared to the HD at both time-point (T0: 4.8 [4.8–4.8] and HD group: 320 [124.1–662.0] BAU/mL, respectively, *p* = 0.0004; Tpost: 9.3 [5.0–36.0] and 3590 [1575–10850] BAU/mL, respectively, *p* = 0.0010) (Figure 2B). 

Finally, after stratifying LuT patients according to their immunosuppressive regimen, no significant difference for humoral response was observed at both time-points (T0: 4.8 [4.8–14.2] and 7.2 [4.8–35.6] BAU/mL; Tpost 19.9 [8.2–449.0] and 762.0 [5.2 vs. 1150] BAU/mL).

### 3.3. Evaluation of T-Cell Response against S and N Peptide Libraries 

Overall, all the enrolled LuT patients and HD had no cytokine production after N peptide library stimulation at both time-points. 

**Figure 3:**
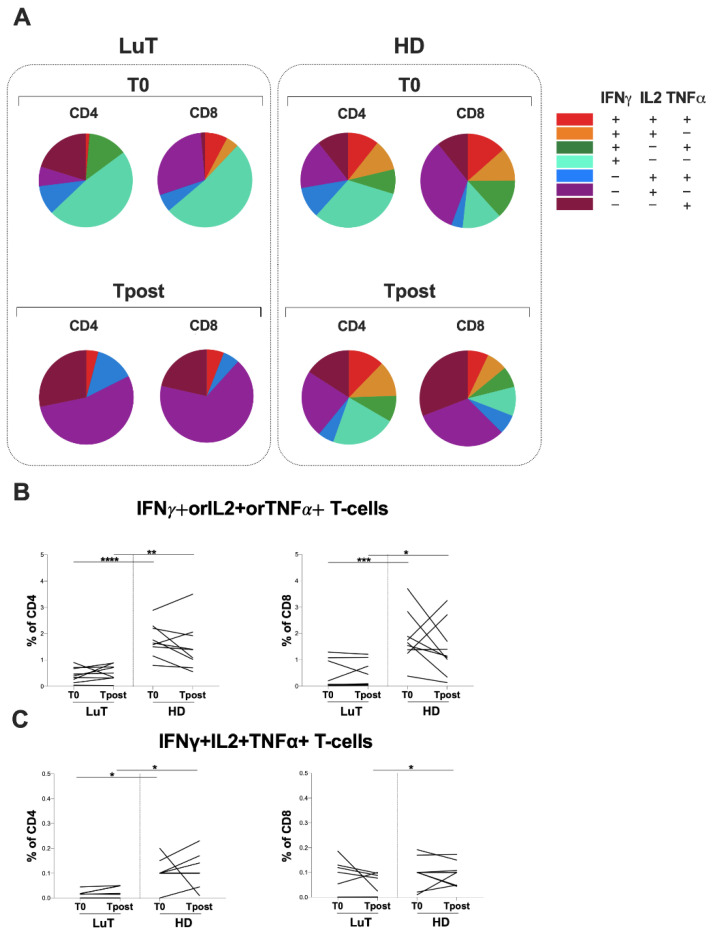
(**A**) Percentages of CD4+ and CD8+ T-cells producing all combinations of IFNγ, IL2, and TNFα by CD4+ and CD8+ T-cells in the LuT and HD groups at both time-points. The fractions of pie charts representing the different combinations of IFNγ, IL2, and TNFα. (**B**) The IFNγ+orIL2+orTNFα+CD4+ and CD8+ T-cell response was evaluated in the LuT and HD groups at two time-points: at T0 and Tpost. Longitudinal evaluation in the LuT group was performed using the Wilcoxon test. Differences between LuT and HD were evaluated using the nonparametric Mann–Whitney test. (**C**) The IFNγ+IL2+TNFα+ CD4+ and CD8+ T-cell response was evaluated in the LuT and HD groups at two time-points: at T0 and at Tpost. Longitudinal evaluation in the LuT group was performed using the Wilcoxon test. The differences between LuT and HD were evaluated using the nonparametric Mann–Whitney test. T0: before the third dose of vaccine; Tpost: two months after the third dose; LuT: lung transplants; HD: healthy donors; ****: *p* < 0.0001; ***: 0.0001 < *p* < 0.001; **: 0.001 < *p* < 0.01; * 0.05 < *p* < 0.01.

After S peptide library stimulation assessed by multiparametric flow cytometry, theT-cell specific response showed an uneven T-cell subset distribution in LuT patients, and this was different between T0 and Tpost (Figure 3A). Conversely, in the HD group, at both time-points, a heterogeneous distribution of T-cell cytokines producers was observed (Figure 3A).

Overall, after comparing each T-cell subset distribution in the LuT patients and HD, at both time-points, the LuT patients showed a significantly lower percentage of IFNγ+IL2+TNFα+CD4+ T-cells and IFNγ+IL2+TNFα-CD4+ T-cells compared with HD (T0: 0.02 [0.00–0.03] and 0.10 [0.10–0.12], *p* = 0.0273; 0.00 [0.00–0.02] and 0.10 [0.06–0.16], respectively, *p* = 0.0007; Tpost: 0.02 [0.00–0.05] and 0.10 [0.07–0.16], *p* = 0.0380; 0.00 [0.00–0.00] and 0.10 [0.02–0.11], respectively, *p* = 0.0013). 

Moreover, at T0, a lower percentage of IFNγ+IL2-TNFα+ CD8+T-cells in LuT patients compared with HD was observed (0.00 [0.00–0.12] and 0.10 [0.06–0.21], respectively, *p* = 0.0305).

At Tpost, in the LuT patients, we found a lower percentage of IFNγ+IL2+TNFα+ CD8+ T-cells (0.03 [0.00–0.10] and 0.10 [0.05–0.13], respectively, *p* = 0.0171), IFNγ+IL2+TNFα-CD8+ T-cells (0.00 [0.00–0.00] and 0.10 [0.05–0.10], respectively, *p* = 0.0003), IFNγ+IL2-TNFα- CD8+ T-cells (0.00 [0.00–0.00] and 0.14 [0.07–0.51], respectively, *p* = 0.00031) and IFNγ-IL2-TNFα+ CD8+ T-cells compared with HD (0.09 [0.01–0.24] and 0.44 [0.09–0.81], respectively, *p* = 0.0423). Finally, at T0, in the LuT patients, a higher percentage of IFNγ+IL2-TNFα-CD8+ T-cells compared with HD was observed (0.36 [0.00–1.25] and 0.10 [0.02–0.75], respectively, *p* = 0.0305). 

At both time-points, the evaluation of T-cells producing IFNγ+orIL2+orTNFα+ showed a lower percentage in the LuT patients compared with HD. This difference was significant for T-cells producing IFNγ+orIL2+orTNFα+ at T0 (CD4: 0.4 [0.2–0.7] and 1.6 [1.3–2.3], respectively, *p* = 0.0001; CD8: 0.1 [0.1–1.0] and 1.7 [1.3–2.4], respectively, *p* = 0.0005) and at Tpost (CD4: 0.5 [0.3–0.81] and 1.4 [0.9–2.0], respectively, *p* = 0.0028; CD8: 0.1 [0.1–0.9] and 1.1 [0.7–2.2], respectively, *p* = 0.0114) (Figure 3B). 

After stratifying LuT patients according to immunosuppressive, treatment no significant difference for T-cell response was observed at T0 (CD4+: 0.0 [0.0–0.2] vs. 0.0 [0.0–0.0] and CD8+: 0.1 [0.0–0.1] vs. 0.0 [0.0–0.2]) and at Tpost (CD4+: 0.0 [0.0–0.2] vs. 0.0 [0.0–0.1] and CD8+: 0.0 [0.0–0.1] vs. 0.0 [0.0–0.1]).

The longitudinal evaluation of the specific T-cell response in LuT patients showed a reduction in the percentage of CD4+ T-cells that produce only IFNγ (IFNγ+IL-2-TNFα-) at Tpost compared with T0 (CD4: 0.0 [0.0–0.2] and 0.6 [0.1–1.1], respectively, *p* = 0.043) (Figure 3A). Finally, we observed a trend in the percentage increase in CD4+ and CD8+ T-cells that produce only TNFα (IFNγ-IL-2-TNFα+) (CD4: 0.1 [0.0–0.8] and 0.2 [0.1–3.5], respectively, *p* = 0.164; CD8: 0.1 [0.0–0.2] and 0.0 [0.0–0.4], respectively, *p* = 0.08) (Figure 3A).

Finally, in LuT patients, no differences in the longitudinal evaluation of IFNγ+orIL2+orTNFα+ and IFNγ+IL2+TNFα+ T-cells were found (Figure 3B,C, respectively).

### 3.4. Evaluation of Spike-Specific T-Cell Response Elicited by Vaccination

**Figure 4:**
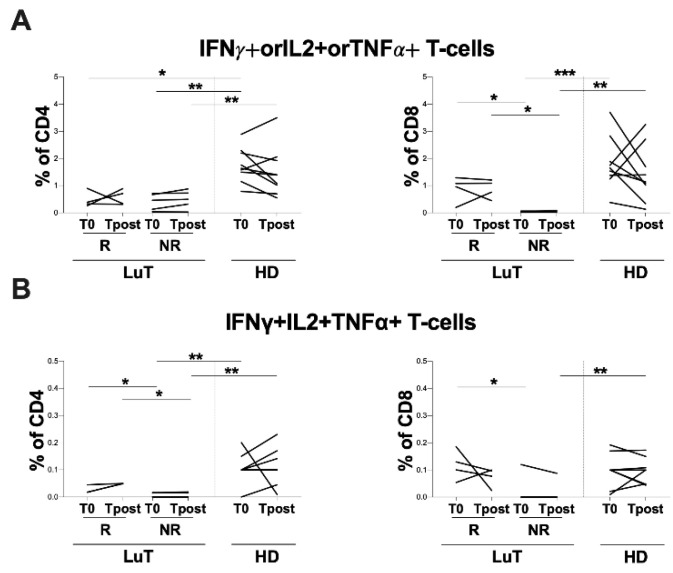
Longitudinal evaluation of T-cell response in LuT patients stratified in the R and NR groups. (**A**) The IFNγ+orIL2+orTNFα+CD4+ and CD8+ T-cell response was evaluated in the R and NR groups. The longitudinal evaluation in each LuT subgroup was performed using the Wilcoxon test. The differences between each LuT subgroup and HD were evaluated using nonparametric the Mann–Whitney test. (**B**) The IFNγ+IL2+TNFα+CD4+ and CD8+ T-cell response was evaluated in the R and NR groups. Longitudinal evaluation in each LuT subgroup was performed using the Wilcoxon test. The differences between each LuT subgroup and HD were evaluated using the nonparametric Mann–Whitney test. T0: before the third dose of vaccine; Tpost: two months after the third dose; LuT: lung transplants; HD: healthy donors; R: responders; NR: non-responders, ***: 0.0001 < *p* < 0.001; **: 0.001 < *p* < 0.01; * 0.05 < *p* < 0.01.

A significant difference in T-cell response was found by stratifying the LuT group according to the humoral response at T0 into R and NR. Specifically, the evaluation of T-cell response, upon stimulation against the Spike-antigen, showed a lower percentage of IFNγ+orIL2+orTNFα+CD4+ and CD8+ T-cells in the NR group compared with the HD group at T0 (IFNγ+orIL2+orTNFα+CD4: 0.5 [0.1–0.7] and 1.6 [1.3–2.3], respectively, *p* = 0.0045; IFNγ+orIL2+orTNFα+CD8: 0.05 [0.03–0.06] and 1.7 [1.3–2.4], respectively, *p* = 0.0008) (Figure 4A) and at Tpost IFNγ+orIL2+orTNFα+CD4+: 0.5 [0.2–0.8] and 1.4 [0.9–2.0], respectively, *p* = 0.0233; IFNγ+orIL2+orTNFα+CD8+: 0.1 [0.0–0.1] and 1.1 [0.7–2.2], respectively, *p* = 0.0159) (Figure 4A).

Moreover, at T0, the NR group showed a lower percentage of IFNγ+IL2+TNFα+CD4+ T-cells compared with HD (0.0 [0.0–0.0] and 0.1 [0.1–0.1], respectively, *p* = 0.0064), as well as at Tpost (0.0 [0.0–0.0] and 0.1 [0.0–0.1], respectively, *p* = 0.0064) (Figure 4B). Conversely, at Tpost, a lower percentage of IFNγ+IL2+TNFα+CD8+ T-cells in the NR group compared with HD was observed (0.0 [0.0–0.0] and 0.1 [0.0–0.1], respectively, *p* = 0.0081) (Figure 4B).

The percentage of IFNγ+IL2+TNFα+CD4+ and CD8+ T-cell response was lower in the NR compared with R at T0 (IFNγ+IL2+TNFα+CD4+: 0.0 [0.0–0.0] and 0.0 [0.0–0.4], respectively, *p* = 0.0159; IFNγ+IL2+TNFα+ CD8+: 0.0 [0.0–0.1] and 0.1 [0.1–0.2], respectively, *p* = 0.0317), whereas at Tpost, only IFNγ+IL2+TNFα+CD4+ was lower in NR compared with the R group (0.0 [0.0–0.0] and 0.0 [0.0–0.5], respectively, *p* = 0.0159) (Figure 4B). 

Finally, at T0, the LuT patients showed a positive correlation between IFNγ+IL2+TNFα+CD4+ and CD8+ T-cells and anti-S IgG titer (ρ = 0.817 *p* = 0.0155; ρ = 0.82 *p* = 0.0175). Moreover, at T0, a negative correlation between percentage of CD4+ T-cells producing only TNFα and the anti-S antibody titer was observed (ρ = −0.733 *p* = 0.0360).

## 4. Discussion

In this observational, monocentric, and prospective study, we investigated the immunogenicity before and after the third dose of BNT162b2 mRNA vaccine in LuT adult patients, evaluating both the humoral and specific T-cell response.

In line with other studies involving different SOT patients [16,17], the first main result of our study was a low serologic response against SARS-CoV-2 both before and after the third dose of BNT162b2 mRNA vaccine in LuT patients compared with HD. Specifically, in line with Tobudic et al. [18], before the third dose, almost all of the LuT patients showed an anti-S antibody titer under the positivity cut-off value (33.8 BAU/mL). Conversely, after the third dose, we observed a clear tendency towards a better humoral response, although it was still significantly lower compared with HD. Overall, our data are in line with other authors [18,19,20], showing an almost complete lack of anti-SARS-CoV-2 antibody response in LuT patients before the booster dose. On the other hand, as reported in a recent work by Catry et al. [21], the third dose induces an increase in the seroconversion rate, underlining its potential benefit, at least within two months from vaccine boosting.

Regarding immunosuppressive therapy, a reduced immune response was mostly reported in patients treated with MMF or rituximab [22,23,24]. MMF, one of the key immunomodulators used in anti-rejection regimens for SOT patients, was associated with weak antibody responses to COVID-19 vaccines [5,9,25] and after conventional vaccination [23]. However, stratifying all the enrolled LuT patients according to MMF therapy, no differences in the anti-SARS-CoV-2 antibody and specific T-cells response were observed. Demographic factors, time since transplantation, and differences in immunosuppressive regimens employed, could probably explain our result.

Although the first line of protection against SARS-CoV-2 includes pre-existing antibodies, induced by vaccination or infection, great safeguards can also be attributed to the T-cell response [16,26]. Indeed, as shown by Agrati et al. [27], in immunocompetent subjects, the BNT162b2 mRNA vaccine is able to elicit a coordinated spike-specific T-cell response characterized by the production of all Th1 cytokines, with IFNγ correlating with both TNFα and IL2. Cellular immunity after COVID-19 vaccination probably plays a contributing role in controlling SARS-CoV-2 infection, even in the absence of a humoral response [22]. Given this, we performed a broad characterization of the functional profiles of specific CD4+ and CD8+ T-cells in LuT patients, and compared the observed findings with HD.

In the LuT patients, we observed an imbalance in T-cell subset distribution at both time-points. Conversely, in the HD group, at both time-points, a heterogeneous distribution of T-cell subsets that produced cytokines was observed. 

Overall, in LuT patients, we observed a lower percentage of IFNγ+orIL2+orTNFα+ and IFNγ+IL2+TNFα+ T-cell response compared with HD. Otherwise, a higher percentage of IFNγ+IL2-TNFα-CD8+ T-cells in LuT patients compared with HD was observed. This is in line with the results from Picchianti-Diamanti et al. [19], showing the production of only one cytokine by T-cells in fragile patients and suggesting a potential disfunction in T-cell response in frail subjects.

Next, stratifying LuT patients according to the lowest cut-off value of detection of humoral response (4.81 BAU/mL) at T0, into R and NR groups, no significant differences in the percentage of IFNγorIL2orTNFα and IFNγ+IL2+TNFα+ T-cell response in the R group compared with HD at both time-points were observed. Otherwise, a lower percentage of IFNγorIL2orTNFα and IFNγ+IL2+TNFα+ T-cell response in the NR group compared with the R group and HD at both time-points was observed. These data seem to confirm that some LuT patients can mount cellular responses even in the absence of a positive humoral response (>33.8 BAU/mL), although this cellular response is dysfunctional, and possibly partially detrimental. Further studies with a higher number of LuT patients are needed to confirm our findings. 

Nevertheless, as reported by Callaghan et al. [28], in a real-world vaccine effectiveness study in SOT patients, SARS-CoV-2 vaccines reduced the risk of death from COVID-19 compared with unvaccinated SOT patients, although the level of vaccine-enabled protection in SOT recipients was markedly less than that observed in the general population.

Our study detected no breakthrough infection in LuT patients up to 2 months from the third dose. This could be explained by a T-cell response in the absence of humoral responses, and by more conscious use of non-pharmacological protective measures. Indeed, strong evidence has shown that routine masking protects against the spread of respiratory viruses, including SARS-CoV-2 [29]. However, more research is needed to determine the benefit of universal masking for SOT patients. 

Our study has some limitations, such as the small sample size due to the peculiarity and high sensibility of lung transplants, as well as the absence of an in-depth investigation into the influence of immunosuppressive therapy on cellular responses. Moreover, we used S-peptide from the Wuhan SARS-CoV-2 strain for the stimulation of T-cells, although the Omicron variant was the dominant circulating strain. However, in terms of the humoral response, Kumar et al. [30] showed that three doses of mRNA vaccine resulted in poor neutralizing responses against the Omicron variant in LuT patients, suggesting the same trend for T-cell response. Further studies are needed to evaluate specific T-cell response against the Omicron variant.

In summary, our data indicate that LuT patients receiving three doses of the SARS-CoV-2 mRNA vaccine had lower humoral and cellular immune responses compared with HD. Overall, our data underline the possibility that some LuT patients can mount cellular responses even in the absence of seroconversion, even if they are lower, dysfunctional, and possibly partially detrimental. Given this, as suggested for other fragile subjects by several authors [20,30,31,32,33], early intervention with monoclonal antibody treatment (such as tixagevimab and cilgavimab) could reduce the risk of COVID-19 severity in LuT patients and could be a useful preventive strategy. 

## Figures and Tables

**Table 1:** Baseline characteristics of the enrolled LuT patients.

Variable	
Age, median (IQR) years	56.0 (46.0–61.5)
Male/Female	7/2
Months since transplant, median (IQR)	24.0 (24.0–102.0)
Immunosuppression treatment	
Steroids	9/9
Antimetabolites	6/9
Calcineurin inhibitors	9/9
Laboratory Data	
WBC (10^9^/L)	8.2 (5.4–10.0)
Neutrophils (10^9^/L)	5.1 (3.6–6.7)
Lymphocytes (10^9^/L)	1.4 (1.3–2.6) 243 (145–265)
PLT (10^9^/L)	
Creatinine (mg/dL)	1.3 (1.2–2.1)
Azotemia (mg/dL)	54 (7.7–85.1)
Comorbidities	
Diabetes	4/9
Arterial hypertension	5/9
Dyslipidemia	1/9
Cardiopathy	2/9

LuT: lung transplant patients; n: number; IQR: interquartile range; WBC: whole blood cell; PLT: platelet. Data are reported as median (interquartile range).

## Data Availability

The raw data supporting the conclusions of this article will be made available by the authors, without undue reservation.

## References

[1] Scharringa S., Hoffman T., van Kessel D.A., Rijkers G.T. (2021). Vaccination and Their Importance for Lung Transplant Recipients in a COVID-19 World. Expert Rev. Clin. Pharmacol..

[2] Haidar G., Agha M., Bilderback A., Lukanski A., Linstrum K., Troyan R., Rothenberger S., McMahon D.K., Crandall M.D., Sobolewksi M.D. (2022). Prospective Evaluation of Coronavirus Disease 2019 (COVID-19) Vaccine Responses Across a Broad Spectrum of Immunocompromising Conditions: The COVID-19 Vaccination in the Immunocompromised Study (COVICS.). Clin. Infect. Dis..

[3] Myers C.N., Scott J.H., Criner G.J., Cordova F.C., Mamary A.J., Marchetti N., Shenoy K.V., Galli J.A., Mulhall P.D., Brown J.C. (2020). COVID-19 in Lung Transplant Recipients. Transpl. Infect. Dis..

[4] Vietri M.T., Albanese L., Passariello L., D’Elia G., Caliendo G., Molinari A.M., Angelillo I.F. (2022). Evaluation of Neutralizing Antibodies after Vaccine BNT162b2: Preliminary Data. J. Clin. Virol..

[5] Cassaniti I., Gregorini M., Bergami F., Arena F., Sammartino J.C., Percivalle E., Soleymaninejadian E., Abelli M., Ticozzelli E., Nocco A. (2022). Effect of a Third Dose of SARS-CoV-2 MRNA BNT162b2 Vaccine on Humoral and Cellular Responses and Serum Anti-HLA Antibodies in Kidney Transplant Recipients. Vaccines.

[6] Gatti M., Rinaldi M., Bussini L., Bonazzetti C., Pascale R., Pasquini Z., Faní F., Pinho Guedes M.N., Azzini A.M., Carrara E. (2022). Clinical Outcome in Solid Organ Transplant Recipients Affected by COVID-19 Compared to General Population: A Systematic Review and Meta-Analysis. Clin. Microbiol. Infect..

[7] Schrezenmeier E., Rincon-Arevalo H., Stefanski A.-L., Potekhin A., Straub-Hohenbleicher H., Choi M., Bachmann F., Pross V., Hammett C., Schrezenmeier H. (2021). B and T Cell Responses after a Third Dose of SARS-CoV-2 Vaccine in Kidney Transplant Recipients. J. Am. Soc. Nephrol..

[8] Schramm R., Costard-Jäckle A., Rivinius R., Fischer B., Müller B., Boeken U., Haneya A., Provaznik Z., Knabbe C., Gummert J. (2021). Poor Humoral and T-Cell Response to Two-Dose SARS-CoV-2 Messenger RNA Vaccine BNT162b2 in Cardiothoracic Transplant Recipients. Clin. Res. Cardiol..

[9] Havlin J., Svorcova M., Dvorackova E., Lastovicka J., Lischke R., Kalina T., Hubacek P. (2021). Immunogenicity of BNT162b2 MRNA COVID-19 Vaccine and SARS-CoV-2 Infection in Lung Transplant Recipients. J. Heart Lung Transplant..

[10] Lozano-Ojalvo D., Camara C., Lopez-Granados E., Nozal P., Del Pino-Molina L., Bravo-Gallego L.Y., Paz-Artal E., Pion M., Correa-Rocha R., Ortiz A. (2021). Differential Effects of the Second SARS-CoV-2 MRNA Vaccine Dose on T Cell Immunity in Naive and COVID-19 Recovered Individuals. Cell Rep..

[11] Mazzone P.J., Mossad S.B., Mawhorter S.D., Mehta A.C., Mauer J.R. (2004). Cell-Mediated Immune Response to Influenza Vaccination in Lung Transplant Recipients. J. Heart Lung Transplant..

[12] Peled Y., Ram E., Lavee J., Segev A., Matezki S., Wieder-Finesod A., Halperin R., Mandelboim M., Indenbaum V., Levy I. (2022). Third Dose of the BNT162b2 Vaccine in Heart Transplant Recipients: Immunogenicity and Clinical Experience. J. Heart Lung Transplant..

[13] Boyarsky B.J., Werbel W.A., Avery R.K., Tobian A.A.R., Massie A.B., Segev D.L., Garonzik-Wang J.M. (2021). Antibody Response to 2-Dose SARS-CoV-2 MRNA Vaccine Series in Solid Organ Transplant Recipients. JAMA.

[14] Iannetta M., Landi D., Cola G., Campogiani L., Malagnino V., Teti E., Coppola L., Di Lorenzo A., Fraboni D., Buccisano F. (2021). B- and T-Cell Responses After SARS-CoV-2 Vaccination in Patients with Multiple Sclerosis Receiving Disease Modifying Therapies: Immunological Patterns and Clinical Implications. Front. Immunol..

[15] Sauzullo I., Mengoni F., Mascia C., Rossi R., Lichtner M., Vullo V., Mastroianni C.M. (2016). Treatment of Latent Tuberculosis Infection Induces Changes in Multifunctional Mycobacterium Tuberculosis-Specific CD4+ T Cells. Med. Microbiol. Immunol..

[16] Rabinowich L., Grupper A., Baruch R., Ben-Yehoyada M., Halperin T., Turner D., Katchman E., Levi S., Houri I., Lubezky N. (2021). Low Immunogenicity to SARS-CoV-2 Vaccination among Liver Transplant Recipients. J. Hepatol..

[17] Collier A.-R.Y., Yu J., McMahan K., Liu J., Chandrashekar A., Maron J.S., Atyeo C., Martinez D.R., Ansel J.L., Aguayo R. (2021). Differential Kinetics of Immune Responses Elicited by Covid-19 Vaccines. N. Engl. J. Med..

[18] Tobudic S., Benazzo A., Koblischke M., Schneider L., Blüml S., Winkler F., Schmidt H., Vorlen S., Haslacher H., Perkmann T. (2022). Immune Response after MRNA COVID-19 Vaccination in Lung Transplant Recipients: A 6-Month Follow-Up. Vaccines.

[19] Picchianti-Diamanti A., Aiello A., Laganà B., Agrati C., Castilletti C., Meschi S., Farroni C., Lapa D., Najafi Fard S., Cuzzi G. (2021). ImmunosuppressiveTherapies Differently Modulate Humoral- and T-Cell-Specific Responses to COVID-19 MRNA Vaccine in Rheumatoid Arthritis Patients. Front. Immunol..

[20] Agrati C., Di Cosimo S., Fenoglio D., Apolone G., Ciceri F., Ciliberto G., Baldanti F., Costantini M., Giannarelli D., Ippolito G. (2021). COVID-19 Vaccination in Fragile Patients: Current Evidence and an Harmonized Transdisease Trial. Front. Immunol..

[21] Catry E., Favresse J., Gillot C., Bayart J.-L., Frérotte D., Dumonceaux M., Evrard P., Mullier F., Douxfils J., Carlier F.M. (2022). Lung Transplant Recipients Immunogenicity after Heterologous ChAdOx1 NCoV-19—BNT162b2 MRNA Vaccination. Viruses.

[22] Mrak D., Tobudic S., Koblischke M., Graninger M., Radner H., Sieghart D., Hofer P., Perkmann T., Haslacher H., Thalhammer R. (2021). SARS-CoV-2 Vaccination in Rituximab-Treated Patients: B Cells Promote Humoral Immune Responses in the Presence of T-Cell-Mediated Immunity. Ann. Rheum. Dis..

[23] Cordero E., Manuel O. (2012). Influenza Vaccination in Solid-Organ Transplant Recipients. Curr. Opin. Organ Transplant..

[24] Cucchiari D., Egri N., Bodro M., Herrera S., Del Risco-Zevallos J., Casals-Urquiza J., Cofan F., Moreno A., Rovira J., Banon-Maneus E. (2021). Cellular and Humoral Response after MRNA-1273 SARS-CoV-2 Vaccine in Kidney Transplant Recipients. Am. J. Transplant..

[25] Hallett A.M., Greenberg R.S., Boyarsky B.J., Shah P.D., Ou M.T., Teles A.T., Krach M.R., López J.I., Werbel W.A., Avery R.K. (2021). SARS-CoV-2 Messenger RNA Vaccine Antibody Response and Reactogenicity in Heart and Lung Transplant Recipients. J. Heart Lung Transplant. Off..

[26] Messika J., Eloy P., Roux A., Hirschi S., Nieves A., Le Pavec J., Sénéchal A., Saint Raymond C., Carlier N., Demant X. (2021). COVID-19 in Lung Transplant Recipients. Transplantation.

[27] Agrati C., Castilletti C., Goletti D., Meschi S., Sacchi A., Matusali G., Bordoni V., Petrone L., Lapa D., Notari S. (2021). Coordinate Induction of Humoral and Spike Specific T-Cell Response in a Cohort of Italian Health Care Workers Receiving BNT162b2 MRNA Vaccine. Microorganisms.

[28] Callaghan C.J., Mumford L., Curtis R.M.K., Williams S.V., Whitaker H., Andrews N., Lopez Bernal J., Ushiro-Lumb I., Pettigrew G.J., Thorburn D. (2022). Real-World Effectiveness of the Pfizer-BioNTech BNT162b2 and Oxford-AstraZeneca ChAdOx1-S Vaccines Against SARS-CoV-2 in Solid Organ and Islet Transplant Recipients. Transplantation.

[29] Puius Y.A., Bartash R.M., Zingman B.S. (2021). Maintaining Mask Momentum in Transplant Recipients. Transpl. Infect. Dis..

[30] Kumar D., Hu Q., Samson R., Ferreira V.H., Hall V.G., Ierullo M., Majchrzak-Kita B., Hardy W., Gingras A., Humar A. (2022). Neutralization against Omicron Variant in Transplant Recipients after Three Doses of MRNA Vaccine. Am. J. Transplant..

[31] Yetmar Z.A., Beam E., O’Horo J.C., Ganesh R., Bierle D.M., Brumble L., Seville M.T., Razonable R.R. (2021). Monoclonal Antibody Therapy for COVID-19 in Solid Organ Transplant Recipients. Open Forum Infect Dis.

[32] Sarrell B.A., Bloch K., El Chediak A., Kumm K., Tracy K., Forbes R.C., Langone A., Thomas L., Schlendorf K., Trindade A.J. (2022). Monoclonal Antibody Treatment for COVID-19 in Solid Organ Transplant Recipients. Transpl. Infect. Dis..

[33] Yetmar Z.A., Bhaimia E., Razonable R.R., Bierle D.M., Ganesh R., Razonable R.R. (2022). Breakthrough COVID-19 after SARS-CoV-2 Vaccination in Solid Organ Transplant Recipients: An Analysis of Symptomatic Cases and Monoclonal Antibody Therapy. Transpl. Infect. Dis..

